# 

**Published:** 1863-10

**Authors:** 


### CONTENTS.

ART. PAGE

I. Baits for Suicide.—"Lady Audley's Secret" and "Aurora Floyd" . . . . . 585

II. The British Medical Association . . . . . 604

III. On a Peculiar Form of Mental Alienation, caused by the Political and Social Disturbances in France, during 1848. By Dr. Bergeret . . . . . 608

IV. The State of Lunacy in Ireland . . . . . 629

V. The Dublin School of Surgery . . . . . 641

VI. The Sanitary State of the Army in India . . . . . 651

VII. On Partial Responsibility in Lunacy and Nervous Disorders. By Dr. Legrand du Saulle . . . . . 668

VIII. The State of Lunacy in England . . . . . 685

IX. On the Legal Responsibility of Pregnant Women. By William Sedgwick, M.R.C.S., and L.S.A. . . . . . 694

X. Murchison on Fever . . . . . 702

XI. Scutari—March, 1863. By J. N. Radcliffe . . . . . 719

XII. Libertinage . . . . . 728

XIII. Gossip . . . . . 736

XIV. Literary Retrospect . . . . . 746

Foreign Medico-Psychological Literature:

1. Synoptical View of Recent Works on Epilepsy . . 752
2. Observations on the Mental Diseases of Criminals . 758
3. Suicide in the United States . . . . . 763

The *Times* on the Insanity of George III. . . . . . 766

Index . . . . . 769

